# Comparing experimental conditions using modern statistics

**DOI:** 10.3758/s13428-020-01471-8

**Published:** 2020-10-09

**Authors:** Jean-Bernard Martens

**Affiliations:** Eindhoven University of Technology, Eindhoven, The Netherlands

**Keywords:** Interactive statistics, Exploratory statistics, Hypothesis testing, Effect size, *t* test, Likert scales, Confidence intervals, Wilks’ theorem, Empirical likelihood

## Abstract

While the applied psychology community relies on statistics to assist drawing conclusions from quantitative data, the methods being used mostly today do not reflect several of the advances in statistics that have been realized over the past decades. We show in this paper how a number of issues with how statistical analyses are presently executed and reported in the literature can be addressed by applying more modern methods. Unfortunately, such new methods are not always supported by widely available statistical packages, such as SPSS, which is why we also introduce a new software platform, called ILLMO (for Interactive Log-Likelihood MOdeling), which offers an intuitive interface to such modern statistical methods. In order to limit the complexity of the material being covered in this paper, we focus the discussion on a fairly simple, but nevertheless very frequent and important statistical task, i.e., comparing two experimental conditions.

## Introduction

The applied psychology community, including my own field of research in human–computer interaction (HCI), relies heavily on empirical research to validate the claims that they make. This empirical research often involves a mix of qualitative and quantitative methods, and statistics is used to analyse the data generated by the quantitative methods. One of the most frequently occurring tasks is to compare two experimental conditions, where the two conditions for instance correspond to a proposed intervention being absent or present. The quantitative measure being used to compare such conditions can either be objective, such as measuring performance time or number of mistakes made, or subjective, such as letting participants express their assessment on one or more attributes on a (7-point) Likert scale. Such measurements can either be performed by the same participants, who get to experience both conditions (i.e., a within-subject experiment), or by two separate groups of participants, each group experiencing one of the conditions (i.e., an across-subject experiment). We will use this relatively simple experimental setup to identify some important issues with how statistical analyses are currently performed and reported, and to propose concrete ways of making improvements by incorporating more modern statistical methods.

In this paper, we will use example data from the popular book by Andy Field (Field, 2013), as this source also provides example formulations for how to report the statistical analyses that are traditionally performed. We will see that these traditional methods focus on establishing statistical significance, instead of on the more relevant aspect of estimating effect size (Lakens, 2013). One major shortcoming of many traditional methods is that, even in cases where they do report measures of effect size, they do not provide confidence intervals for such measures, so that the accuracy with which such estimates are made is unknown. This accuracy has a direct influence on the strength of the claim that is supported by the data (i.e., there is an obvious difference between claiming a medium-size effect and concluding that the effect is somewhere in between non-existing and huge). In this paper, we will present an alternative approach that is based on the recent theory of multi-model comparisons (Burnham & Anderson, 2002), and more specifically Wilks’ theorem (Wilks, 1938), which provides a solution to such problems. As several of the analyses that we promote in this paper are likely to be unfamiliar for many psychologists, we also propose formulations for how to report such new types of analyses in scientific publications.

A second aspect that can be improved in current statistical practice is the use of non-parametric methods. Such methods are used when the assumption of normality, which is implicit in popular statistical methods such as the *t* test, is violated. Non-parametric methods, such as Wilcoxon tests (Field, 2013), rely on the assumption that a rank-order transformation on the data results in transformed data that are approximately normally distributed. There is however no guarantee that this will indeed be the case (as can easily be established through a counter example). We will therefore introduce as an alternative the method of empirical likelihood (EL) (Owen, 2001) which does not rely on such a questionable assumption. An important advantage is that Wilks’ theorem can be extended to empirical likelihood, so that EL cannot only be used to test significance, as the existing non-parametric methods do, but also allows to estimate confidence intervals in a way that is very similar to how it is done in the case of parametric methods. Such a uniform treatment of both parametric and non-parametric statistics is an obvious advantage, as users need to acquaint themselves with fewer distinct principles.

A third issue that has been discussed earlier in the literature is how to analyse discrete ordinal data (Kaptein, Nass, & Markopoulos, 2010; Robertson & Kaptein, 2016), such as gathered through questionnaires with Likert scales, in a theoretically sound way. We will show how a 100-year-old method, called Thurstone modelling (Thurstone, 1927), can be used to model such discrete ordinal data by means of continuous distributions. By doing so, the data analysis for ordinal data can be closely aligned with the (parametric) data analysis for continuous data, as the theory of multi-model comparisons and Wilks’ theorem apply equally to both kinds of data.

Introducing new statistical methods would be of limited practical interest if such methods would not be accessible to the empirical researchers that need them, preferably in a user-friendly way, i.e., without the need for extensive programming from the side of these researchers. We will show how ILLMO, a program that allows to perform statistics in an interactive way, supported by ample graphical user interfaces and visualisations, can be used to perform all statistical analyses that are discussed in this paper. An earlier version of this program, which included only some of the features discussed in the current paper, has already been introduced in earlier publications (Martens, 2012; 2014; 2017). It is worthwhile to note that all graphs that are included in this paper to clarify the statistical methods under discussion have actually been generated by the ILLMO program, illustrating that the program not only focuses on conducting statistical analyses, but also on documenting them.

This paper is structured in the following way. In Section “Within-subject analysis (with parametric statistics)”, the focus is on how to analyse data from a within-subject experiment, using example data taken from the book of Andy Field. We use the dependent *t* test as the starting point to argue the need for more advanced statistical parametric methods that focus not only on establishing effect size, but also on estimating the accuracy with which this effect size can be determined from the data. The ability to compare alternative models for the same data, using multi-model comparisons, and the use of confidence intervals for key statistical parameters, such as effect sizes, are introduced and illustrated on the example data. In Section “Across-subject analysis (with parametric statistics)”, a similar discussion is conducted using data from an across-subject experiment, again taken from the book of Andy Field. Next, in Section “Interactive statistics with ILLMO”, we demonstrate how the theoretical methods for parametric statistics, introduced in the two previous sections, have been implemented into a new interactive program for statistical analysis, called ILLMO. Some aspects of the interface that relate to the statistical comparison of two experimental conditions are explained in detail, while a reference is made to the ILLMO project website for an explanation of additional statistical methods, such as simple and multiple regression.

In Section “Non-parametric statistics”, we move back to theoretical concepts, more specifically to the issue of non-parametric statistical analyses.

Specifically, we show how the method of empirical likelihood provides an approach to estimating effect sizes and their confidence intervals that is very similar to the approach taken in Sections “Within-subject analysis (with parametric statistics)” and “Across-subject analysis (with parametric statistics)” for parametric models. Existing methods for performing empirical likelihood estimation of confidence intervals for averages, moments and quantiles (including medians), which are included in the statistical programming language R, have been made available through the graphical user interface of ILLMO. While ILLMO has been offering some empirical likelihood methods, such as the non-parametric estimation of receiver operating characteristics, that were previously not included in R, recent extensions to empirical likelihood estimations in R (Chaussé, 2010) now also offer methods in R that are not covered in ILLMO.

In Section “Analysing discrete (ordinal) data”, we conclude the theoretical treatment by discussing how an age-old method, introduced by Thurstone in the 1920s, has been used in ILLMO to analyse discrete ordinal data, as for instance collected through questionnaires on subjective impressions. While some classes of Thurstone modelling can also be executed in R, the ILLMO program offers such methods though a user-friendly graphical user interface. In the concluding Section “Conclusions”, we discuss how the methods discussed in this paper and their implementation in ILLMO help to address several of the statistical challenges raised by Kaptein and Robertson (for the computer–human interaction community) (Kaptein & Robertson, 2012).

## Within-subject analysis (with parametric statistics)

In chapter 9 of the book “Discovering Statistics using SPS” (Field, 2013), entitled “Comparing two Means”, Andy Field introduces a simple data set that is reproduced in Table 1. The participants in the experiment were asked to fill in an anxiety questionnaire which was used to generate a single dependent variable, i.e., the anxiety scores shown in the table. The two central columns in the table correspond to the two experimental conditions, which result from changing the independent variable, either confronting the participants with the picture of a spider or with a real tarantula. The research question is whether or not there is an effect of the independent variable on the dependent variable, and to estimate the effect size.

**Table 1:** Anxiety scores in response to the picture of a spider or a real spider (from Field, 2013)

Subject	Picture	Real	Real-Picture
1	30	40	10
2	35	35	0
3	45	50	5
4	40	55	15
5	50	65	15
6	35	55	20
7	55	50	-5
8	25	35	10
9	30	30	0
10	45	50	5
11	40	60	20
12	50	39	-11

### Traditional statistics

Field describes how to perform a *dependent* *t* *test* on the data, which corresponds to assuming that the data resulted from a within-subject experiment. This test uses the difference scores for all participants, shown in the last column of Table 1, and determines whether or not the average across participants of these differences in anxiety scores deviates from zero. The outcome of the dependent *t* test was summarised as follows by Field: *On average, participants experienced significantly greater anxiety to real spiders* (*mean* = 47,*se* = 3.18) than to pictures of spiders (*mean* = 40,*se* = 2.68), *t*(11) = 2.47, *p* = 0.031(< 0.05), with effect size *R* = 0.60. The *t* test hence establishes that an observed *average difference* of *dif* = 7 or higher between both conditions is unlikely to occur, more specifically, with probability equal to *p* = 0.031, in case the *population average for this difference is equal to zero*, which is the assumption made by the so-called *null hypothesis* *H*_0_ that is being tested by the *t* test.

It is by now well established within the applied psychology community that only reporting significance is insufficient, as significance is not only a property of the phenomenon being studied, but also depends on the size of the sample being used in the experiment (*N* = 12 participants in the experiment under study). Otherwise stated, as the number of participants increases, the average difference is likely to stay approximately the same, but the T-statistic will increase, so that it can be expected to supersede the threshold for significance once the number of participants is large enough. This has led the American Psychological Association (APA) (Cumming, Fidler, Kalinowski, & Lai, 2012) to recommend that at least one measure of *effect size* should be included when reporting experimental findings. In the previous quote from the book of Andy Field, the chosen measure of effect size is the (Pearson) correlation coefficient of *R* = 0.60. This correlation coefficient is not explicitly provided by SPSS but can be derived by means of the simple formula

1 $$ R^{2} = \frac{t^{2}}{t^{2}+df}, $$

where *df* denotes the number of degrees of freedom in the *t* test, which is *df* = *N* − 1 = 11 in case of the reported experiment. Note that, as *t*^2^ is expected to increase approximately linearly with *df*, this correlation coefficient *R* does not have the dependence on the sample size *N* that the T-statistic *t* itself has. The squared correlation *R*^2^ is the fraction of variation in the difference scores that can be attributed to the average difference, the remaining part being the fraction of variation around this average difference. The correlation coefficient is a number between 0 and 1, where zero corresponds to no effect at all, and one corresponds to a very large effect. It is, however, not very intuitive how to interpret intermediate values, nor is it clear if we should prefer *R* over *R*^2^ as a measure of effect size? This may explain why the correlation coefficient is not widely used as a measure of effect size when comparing experimental conditions, which is why we will provide some more popular (and intuitive) alternatives below.

The interactive statistical program ILLMO that we will introduce in more detail later on in the paper also allows to perform a dependent *t* test and provides additional information that can be used to make some more observations on the *t* test, i.e.,


Gaussian model - dif = 7 Planned test (variance is estimated): sd (standard deviation) = 6.93476 se_dif (standard error of dif)= 2.8311 Effect sizes: Cohen's d = |dif|/sd = 1.009408 JND = 0.713759 (medium effect) probability of superiority P(dif>0) = 0.762312 Hedgens' g = 0.850577 (g/d = 0.842650) JND = 0.601449 (medium effect) probability of superiority P(dif>0) = 0.726230 squared correlation: R2 = 0.35723 correlation: R = 0.597687 confidence CI(R) = [0.069638,0.864106] t test (two-sided) for equal average: T(11) = dif/se_dif = 2.4725 (p=0.0309818) |T| >= 2.2010 (p=0.05) : significant confidence CI(dif) = [0.768763,13.2312] Estimated power is 0.607013(need 1.98 times more trials for power 0.8)

We can observe in this output that the *t* test is equivalent to estimating a 95% confidence interval (CI) for the average difference, equal to CI(dif)=[0.77,13.23] for the current experiment. The interpretation of this CI is that *the probability that the true population value for the average difference lies within this interval is 95%*. The *t* test is significant because the zero value, which corresponds to the null hypothesis *H*_0_, is outside of this CI, so that the probability that the actual population value for the average difference is indeed equal to zero, as proposed by the null hypothesis, is very low (*p* = 0.03 < 0.05). Note that the *t* test makes clear that this population value can only be estimated with a limited accuracy from an experiment with few participants, hence the relatively large size of the CI. The estimate can be improved, in the sense that the size of the CI can be reduced, by including more participants in the experiment.

This recognition of an inherently limited accuracy when estimating statistical parameters from experimental data was obviously not reflected when we reported the effect size of *R* = 0.60 above. There is, however, a historical reason for this discrepancy, i.e., the fact that there exists an analytical expression, which is used by statistical programs such as SPSS and ILLMO, for the CI of the average difference, but that no such expression exists for most effect sizes. Nevertheless, the ILLMO output also includes a CI for the effect size *R*, more precisely equal to CI(R)=[0.070,0.864] for the current experiment. This CI(R) clarifies that the correlation coefficient R can only be established with very limited accuracy based on an experiment with few participants. The *CI*(*R*) is determined using an approximation technique that can be shown to produce a CI that is asymptotically correct,^1^ i.e., the accuracy of this CI estimate will improve as the number of participants increases. With modern computers it is possible to perform numerical approximations in cases where no analytical expressions are available. They provide information that would otherwise not be available, and which is essential when drawing inferences from effect sizes.

Note that the ILLMO program also offers an alternative indicator for effect size, called *Cohen’s d* (of *d* = 1.009). It is known that Cohen’s *d* is upwardly biased when using small sample sizes, which is why we also report *Hedgen g* (of *g* = 0.85), which is corrected for this bias (Lakens, 2013). Provided that the differences in anxiety scores can be assumed to be normally distributed, as is the implicit assumption made by the *t* test, these effect sizes can in turn be mapped to another effect size with a more intuitive meaning, i.e., the *probability of superiority*

2 $$ P_{s} = P(dif>0) = P_{n}(JND), $$

where *P*_*n*_ denotes the normalised cumulative Gaussian distribution. We have introduced the *Just Noticeable Difference (JND)*, which is either equal to $JND=d/\sqrt {2}$, in case Cohen’s *d* is used, or equal to $JND=g/\sqrt {2}$, when Hedgens’ g is preferred. This relationship is illustrated in Table 2, together with the subjective associations that are often used in the psychological literature to denote effect sizes. For example, in case of the reported experiment, the *JND* = 0.714 according to Cohen’s d can be characterised as a “medium effect” with a probability of superiority equal to *P*_*s*_ = 0.7623. Note however that such subjective characterisations are area-specific, as a large-sized effect in areas such a psychology may be considered as small in other fields, such as engineering. Therefore, many statisticians do not advertise their use.

**Table 2:** Effect size (in JND) related to the probability of superiority in case of a Gaussian distribution

Subjective	Effect	Probability
Description	Size (in JND)	of Superiority (percentage)
tiny	[0.0, 0.2[	[50.00, 57.93[
small	[0.2, 0.5[	[57.93, 69.15[
medium	[0.5, 0.8[	[69.15, 78.81[
large	[0.8, 1.0[	[78.81, 84.13[
1 JND	1.0	84.13
1.5 JND	1.5	93.32
2 JND	1.0	97.72
2.5 JND	2.5	99.38
3 JND	3.0	99.87

The probability of superiority is a measure for effect size that has an intuitive interpretation, i.e., that *there is an estimated probability of* *P*_*s*_ = 0.762 *for participants to report a higher anxiety score when confronted with a real spider instead of with the picture of a spider*. This is higher than the chance level *P*_*s*_ = 0.5 that is expected when the independent condition would not have any effect (i.e., the null hypothesis *H*_0_). What is currently missing are confidence intervals for the newly introduced measures for effect size, i.e., Cohen’s *d* (or it’s scaled version Hedgens g) and the probability of superiority *P*_*s*_. This will be remedied in the next section through the use of modern statistics, more specifically by applying Wilks’ theorem, and will allow us to add the required nuance to the above conclusion.

Note that the *t* test focuses on identifying instances where there is evidence to reject the null hypothesis, i.e., to reject cases where a change in the experimental condition does not have an effect on the dependent variable. The *t* test does so with a confidence of 1 − *α*, where *α* = 0.05 is the most frequently adopted choice for the *Type I error* (the probability of wrongfully rejecting the null hypothesis, i.e., deciding that there is an effect when there is actually none). The *t* test in ILLMO also determines estimates for the effect size, such as Cohen’s d. In order to avoid that we unjustly conclude that observed significance implies that the experimental condition does have an effect of the size estimated by the *t* test, which is usually referred to as *the alternative hypothesis* *H*_1_, we also need to take into account the *Type II error* *β* (the probability of unjustly rejecting such an effect size). A minimum value of 1 − *β* = 0.8 for the so-called *power* is usually advised in psychological literature. The *t* statistic derived by the *t* test can be used to estimate this power.^2^ More practically, it can also be used to make an educated guess for how many more participants are expected to be needed to reach the prescribed minimum value for the power of *β* = 0.8. This information is also reported in the output from the ILLMO program and constitutes a very practical piece of information for the experimenter. Indeed, as an expert in statistics, one of the most frequently asked questions is how many participants need to be included in an experiment. While this question can usually not be answered in advance, a reasonably accurate guess can be made once the experiment has been executed by a limited number of participants and a (preliminary) estimate can be made of the effect size.

### Modern statistics

Statistical terms such as significance and statistical power are known to be confusing for many non-statisticians interested in empirical research. We therefore offer an alternative interpretation of the *t* test that is based on the recent theory of *multi-model comparisons* (Lindberg, Schmidt, & Walker, 2015; Burnham & Anderson, 2002; Burnham & Anderson, 2004; Burnham, Anderson, & Huyvaert, 2011). Multi-model comparisons use the *Akaike Information Criterion* (AIC) to compute the *likelihood ratio between alternative models* for the same observed data. In case of the *t* test and the experiment under discussion, the first model corresponds to the null hypothesis *H*_0_ and assumes a known value of zero for the average difference in anxiety scores, while the second model corresponds to the alternative hypothesis *H*_1_ and assumes that the average difference is a parameter that is not known a priori and hence needs to be estimated from the data. Both models agree in that the standard error is not known a priori and therefore also needs to be estimated from the data. This results in the first model with zero average difference having one parameter (*P*_1_ = 1) and the second model having two parameters (*P*_2_ = 2).

We will denote the probability distribution that is adopted by model *i* by *p*_*i*_(*x*,*𝜃*_*i*_), where *𝜃*_*i*_ is a vector with *P*_*i*_ parameters, for *i* = 1,2. The AIC can be expressed as

3 $$ AIC_{i} = LLC_{i} + 2 P_{i}\cdot \frac{N}{N-P_{i}-1}, $$

for *i* = 1,2, where the *Log-Likelihood Criterion* (LLC)

4 $$ LLC_{i} = -2 \sum\limits_{j=1}^{N} \log(p_{i}(x_{j},\theta_{i}) ), $$

with {*x*_*j*_, *j* = 1,…,*N*} denoting the observed difference values, is a *measure for the lack-of-fit of the* *i*-*th model to the data* (*LLC*_1_ for model *i* = 1 and *LLC*_2_ for model *i* = 2). The simplest model, i.e., model 1, will typically have an *LLC*_1_ that is larger than the *LLC*_2_ for the more complex model 2. The interesting question is whether or not this improved fit warrants the use of a model with one extra parameter?

The key result from the theory of multi-model comparison (Burnham & Anderson, 2002; Burnham et al., 2011) is that the *likelihood ratio between two models for which the AIC can be computed* is equal to

5 $$ \ell_{21} = \exp\left( -\frac{AIC_{2}-AIC_{1}}{2} \right), $$

where a likelihood ratio of *ℓ*_21_ > 1 implies that the model where the average difference is a parameter (model 2) is more likely than the model where the average difference is a priori assumed to be equal to zero (a value of *ℓ*_21_ < 1 obviously leads to the opposite conclusion).^3^ The program ILLMO can convert many traditional statistical tests, including the *t* test, into AIC values, following a procedure that is discussed in detail in (Burnham & Anderson, 2002), and can subsequently perform the corresponding multi-model comparison. The output from the ILLMO program for our example data is the following:


Multi-model comparison: Model 1: equal averages (P=1,LLC=33.4711) Model 2: unequal averages (P=2,LLC=28.1675) Model 1: AIC = 35.8711 Model 2: AIC = 33.5008 Model 2 has lowest AIC Model likelihoods (L) and weights (W) Model 1: L = 0.305703, W = 0.234129 Model 2: L = 1, W = 0.765871

This implies that the more complex model is *ℓ*_21_ = 1/0.305703 = 3.27 times more likely than the simpler model. In order to help with interpreting this likelihood ratio, the subjective scale proposed by Jeffreys (Jeffreys, 1961; Robert, Chopin, & Rousseau, 2009), which is summarised in Table 3, can be used. This for instance leads to the following way of reporting the multi-model comparison: *A model that assumes that participants on average experience a different anxiety to real spiders than to pictures of spiders is* *ℓ*_21_ = 3.27 *times more likely than a model in which participants are assumed to experience the same level of anxiety; such a likelihood ratio can be characterised as “substantial” evidence in favour of the most likely model*.

**Table 3:** Subjective scale for classifying the likelihood ratio, proposed by Jeffreys

AIC increase	Likelihood	Likelihood	Strength of
		Ratio	Evidence
0 to 2.3	> 1.0000	> 1	barely worth mentioning
2.3 to 4.6	< 0.3165	> 3.16	substantial
4.6 to 6.9	< 0.1000	> 10	strong
6.9 to 9.2	< 0.0317	> 31.6	very strong
> 9.2	< 0.0100	> 100	decisive

**Fig. 1:**
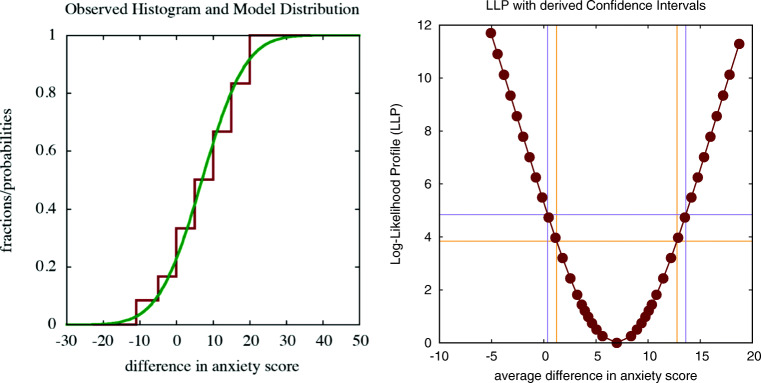
The stepwise curve in the left figure shows the observed cumulative histogram for the differences in anxiety scores, while the continuous curve is the best fitting Gaussian distribution that is used to model it. The figure on the right shows the log-likelihood profile (LLP) for the average difference, intersected at two levels (corresponding to a ${\chi _{1}^{2}}$ and an *F*_1,11_ criterion) to establish two distinct but similar estimates for the confidence interval (CI) of the average difference (spider data: parametric within-subject analysis)

Traditional hypothesis testing can be considered as a special case of multi-model comparison. For instance, the dependent *t* test fits a Gaussian model to the observed histogram of differences in anxiety scores, as shown on the left in Fig. 1. Such a model has two parameters, the average and the standard deviation of the Gaussian distribution. The optimal values for these parameters are obtained by minimising a criterion such as the LLC that expresses the lack-of-fit between the distribution and the observed histogram. For the experiment under discussion, the optimal estimate for the average difference of *dif* = 7 is the same as in case of the *t* test. The optimal value for the standard deviation *σ* = 6.639 is however slightly different from the value of the standard deviation *sd* = 6.935 reported in the *t* test, as the LLC used in the multi-model comparison is slightly different from the optimisation criterion used in the *t* test. This translates into values for Cohen’s *d* = 1.054, *JND* = 0.745 and the probability of superiority of *P*_*s*_= 0.772 that also deviate slightly from those reported earlier.

A hypothesis corresponds to an assumption about one of the distribution parameters; for example, in case of the dependent *t* test the assumption in the null hypothesis under test is that the average of the Gaussian distribution is equal to zero. The ILLMO program can focus on one such distribution parameter and can calculate the so-called *log-likelihood profile* (LLP), which is a function that expresses how the lack-of-fit criterion (in our case, the LLC) increases for values of the parameter that are different from the optimal value (Uusipaikka, 2009). On the right in Fig. 1, we show the LLP for values of the average difference in anxiety scores that are close to the optimal value of *dif* = 7.

There is an important result in statistics, called *Wilks’ theorem* (Wilks, 1938), that establishes a relationship between such an LLP and a classic hypothesis test. More precisely, this theorem states that an (asymptotically correct) estimate for the confidence interval for the parameter in the LLP can be obtained by intersecting the LLP at a level that is determined by the required level of confidence (Meeker & Escobar, 1995). More specifically, intersecting at the chi-squared value with one degree of freedom, i.e., ${\chi _{1}^{2}}(1-\alpha =0.95)=3.84$, is most frequently used to construct the 95% confidence interval. This results in a 95% confidence interval equal to CI(dif)=[1.24,12.77] for the average difference in anxiety scores, which is slightly smaller than the estimate of CI(dif)=[0.77,13.23] found earlier using the *t* test (which serves as a reference as it is based on an exact analytical expression). An alternative proposal, which is assumed to be more accurate in case the number of observations *N* is small, is to intersect the LLP at the value of the F-distribution with (1,*N* − 1) degrees of freedom, i.e., *F*_1,11_(1 − *α* = 0.95) = 4.84, which results in confidence interval equal to CI(dif)=[0.38,13.62] for the average difference in anxiety scores, which is slightly larger than the CI found using the *t* test. The two levels will converge if the number of participants *N* increases, as $F_{1,N-1}(1-\alpha )\rightarrow {\chi _{1}^{2}}(1-\alpha )$ if $N\rightarrow \infty $.

**Fig. 2:**
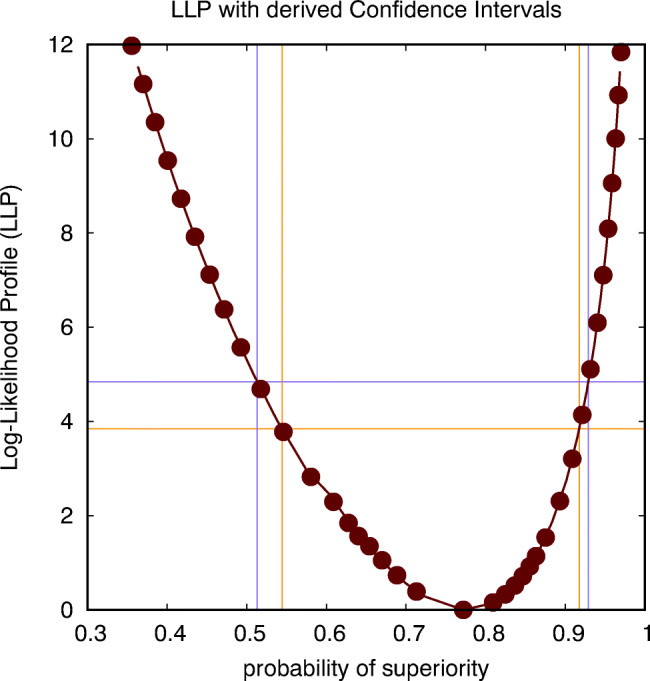
The log-likelihood profile (LLP) for the probability of superiority, intersected at two levels (corresponding to a ${\chi _{1}^{2}}$ criterion and an *F*_1,11_ criterion) to establish two distinct but similar estimates for the confidence interval (CI) of this probability (spider data: parametric within-subject analysis)

There is obviously little need to use an approximate method if we can assume that a Gaussian distribution is a good model for the observed histogram and we are pursuing a CI for the mean value, as an exact (analytical) expression is known for this case. However, if a different distribution is more appropriate to model the observed histogram or if the CI for another parameter than the mean is required, using the above approximation may be the only option available. More specifically, we will rely on such approximate methods in case we need the CI for measures of effect size, such as Cohen’s *d* or the probability of superiority *P*_*s*_. In Fig. 2, we show the LLP for the probability of superiority, resulting in a CI(*P*_*s*_)=[0.544,0.917] in case the intersection is performed at a level of 3.84, and CI(*P*_*s*_)=[0.513,0.929] in case the intersection is performed at a level of 4.84. These estimates allow us to complete the report on the statistical analysis that we proposed earlier, i.e., that *there is an estimated probability of* *P*_*s*_= 0.772, *with a 95% confidence interval equal to* CI(*P*_*s*_)=[0.544,0.917], *according to a *${\chi _{1}^{2}}$ *criterion, or* CI(*P*_*s*_)=[0.513,0.929], according to an *F*_1,11_ *criterion, for participants to report a higher anxiety score when confronted with a real spider instead of with the picture of a spider*. Note that significance corresponds to the fact that the chance level (*P*_*s*_ = 0.5) is outside of these estimated CIs. According to Table 2 the effect size is hence in between tiny and 1.5 JND, which is quite a large range because the number of observations is rather limited.

One remaining issue that hasn’t been addressed yet is whether or not the assumption of a Gaussian distribution, which is implicit in both the dependent *t* test and the construction of the LLP, is indeed valid. There are tests for normality (such as the K-squared test of d’Agostino which is used within the ILLMO program, or the Kolmogorov–Smirnov and Shapiro–Wilk tests offered by SPSS (Field, 2013)) that can test whether or not an observed histogram deviates significantly from a Gaussian distribution. They however offer little or no advice on how to proceed in case the test fails, except maybe to adopt a non-parametric procedure, as described in a later section. In cases where there is a lack-of-fit between an observed histogram and the optimal Gaussian distribution, or in cases where we a priori expect that other distributions may be better suited, multi-model comparison can come to the rescue, as it can be used to establish the likelihood for each of the alternative (parametric) distributions being considered. For instance, for our example data, a Gaussian distribution is estimated to be *ℓ* = 3.93 times as likely as a Laplacian distribution, which constitutes substantial evidence in favour of the Gaussian distribution over the Laplacian distribution. However, a Student-T distribution (with 4 degrees of freedom) is estimated to be *ℓ* = 15.19 times as likely as a Gaussian distribution, which constitutes strong evidence in favour of the Student’s *t* distribution over the Gaussian distribution. This could for instance lead to the following alternative summary of the analysis: *the histogram of observed differences in anxiety scores can be modelled by Student’s* *t* *distribution with 4 degrees of freedom; this model predicts a probability of* *P*_*s*_= 0.790 , *with a 95% confidence interval equal to CI*(*P*_*s*_)=[0.549,0.914], *according to a *${\chi _{1}^{2}}$ *criterion, or CI*(*P*_*s*_)=[0.515,0.923], *according to an* *F*_1,11_ *criterion, for participants to report a higher anxiety score when confronted with a real spider instead of with the picture of a spider*.

Note that, despite the fact that there is strong evidence that the Student’s *t* distribution is a better model than the Gaussian distribution, the LLP and hence also the CIs derived from it, turn out to be fairly robust against such changes in the model. This is probably due to the fact that both proposed distributions provide reasonably good approximations to the observed histogram. This observation reassures us that reasonable goodness-of-fit seems to be enough to obtain reliable estimates for CIs.

## Across-subject analysis (with parametric statistics)

### Traditional statistics

Andy Field also used the data in Table 1 to illustrate the *independent* *t* *test*, which corresponds to assuming that the data resulted from an across-subject experiment. This assumes that the anxiety scores in the same row in Table 1 were not generated by the same participant experiencing both conditions, but by separate participants in distinct conditions, which in turn implies that the analysis can no longer focus on the average of the differences in anxiety scores, but instead needs to compare the averages of the anxiety scores themselves. The result of the independent *t* test analysis was reported as follows by Field: *On average, participants experienced greater anxiety to real spiders* (mean= 47, se= 3.18) *than to pictures of spiders (mean= 40, se= 2.68); this difference was not significant* *t*(22) = 1.68, *p* > 0.05, *however, it did represent a medium-sized effect of* *R* = 0.34.

The statistical program ILLMO can also perform the independent *t* test and provides additional information that can be used to make some more observations on the *t* test, i.e.,


Gaussian model : dif = 7 Planned test (assume equal variance): sd (pooled standard deviation) = 10.198 se_dif (standard error of dif) = 4.16333 Effect sizes: Cohen's d = |dif|/se = 0.686406 JND = 0.485363 (small effect) probability of superiority P(dif>0) = 0.686290 Hedgens' g = 0.634523 (g/d = 0.924412) JND = 0.448675 (small effect) probability of superiority P(dif>0) = 0.673167 squared correlation: R2 = 0.113865 correlation: R = 0.337439 confidence CI(R) = [-0.076347,0.652072] F-test for equal variance: sd(stimulus 2) [12 trials] = 11.0289 sd(stimulus 1) [12 trials] = 9.2932 F(11,11) = (sd_2/sd_1)**2 = 1.4084 (p=0.289837) F < 2.8178 (p=0.05) : equal variance t test (two-sided) for equal average: T(22) = dif/se_dif = 1.6813 (p=0.106839) |T| < 2.0739 (p=0.05) : not significant confidence CI(dif) = [-1.63432,15.6343] Estimated power is 0.347326 (need 3.98 times more trials for beta=0.8)

We can observe in this output that the *t* test is equivalent to estimating a 95% confidence interval for the difference in averages, equal to CI(dif)=[-1.63,15.63] for the current experiment. The *t* test is not significant because the zero value, which corresponds to the null hypothesis *H*_0_, is inside of this CI. The output also reports on the result of the *F-test for equal variance* which supports the choice for an independent *t* test that assumes equal variance in both conditions.

The estimated CI for the effect size of *R* = 0.34 is CI(R)=[-0.076,0.652], which means that we can not exclude with 95% confidence that there is no effect at all (which corresponds to zero correlation). Alternative indicators for the effect size are Cohen’s *d* = 0.686, with *JND* = 0.485 and probability of superiority *P*_*s*_= 0.686, and Hedgens’ *g* = 0.635, with *JND* = 0.449 and probability of superiority *P*_*s*_= 0.671. According to Table 2, this effect is close to the boundary between a small and a medium effect. Note that the reported probability of superiority is lower than in case of the dependent *t* test, which agrees with our intuition that personal differences in how anxiety is scored affect the reliability of the anxiety scores in an across-subject experiment, while a within-subject experiment can account for some of these personal differences by only relying on differences in anxiety scores. This reduced effect size is also reflected in how many more participants are expected to be required to be able to conclude, with a minimal power of *β* = 0.8, that there indeed exists an effect of the size that has been estimated. This factor of 3.98 for the across-subject experiment is about two times as large as the factor of 1.98 found earlier for the within-subject experiment. This means that the latter experiment is more powerful, as fewer measurements are needed to reach the same level of accuracy (i.e., type II errors).

The above results allow us to conclude (using Cohen’s *d*) that *there is an estimated probability of* *P*_*s*_ = 0.686 *for participants to report a higher anxiety score when confronted with a real spider instead of with the picture of a spider*. Of course, as we do not know the CI for this probability of superiority, it is unclear (at this time) what we can actually conclude from this statement. The next section will address this issue.

### Modern statistics

Similarly as in the case of the within-subject analysis, we can perform a multi-model comparison in case of an across-subject analysis. The main difference is that both conditions are now modelled by a separate distribution (*p*_*i*_(*x*,*𝜃*_*i*_) for condition *i* = 1,2) and that there is a separate log-likelihood criterion (LLC) to express the lack-of-fit in each of the two conditions, i.e.,

6 $$ LLC_{i} = -2 \sum\limits_{j=1}^{N_{i}} \log(p_{i}(x_{ij},\theta_{i}) ), $$

where {*x*_*ij*_, for *j* = 1,…,*N*_*i*_} are the observed values in condition *i*, for *i* = 1,2. The total number of observations is *N* = *N*_1_ + *N*_2_. The LLC for the complete experiment including both conditions is obtained by adding the LLC values for each of these conditions. This total *LLC* = *LLC*_1_ + *LLC*_2_ can be combined with the total number of parameters *P* (across all conditions) to define the AIC in exactly the same way as before.

We assume a first model, corresponding to the null hypothesis *H*_0_, where the averages of the distributions in both conditions are the same, which leads to one parameter for this shared average. We assume a second model , corresponding to the alternative hypothesis *H*_1_, where the averages of the distributions in both conditions are distinct, resulting in two parameters, one for each of the average values. Both models assume the same standard deviation for both conditions, which adds one parameter to both models. The output from the ILLMO program for the multi-model comparison is:


Multi-model comparison: Model 1: equal averages (P=2,LLC=61.1119) Model 2: unequal averages (P=3,LLC=58.2106) Model 1: AIC = 65.6833 Model 2: AIC = 65.4106 Model 2 has lowest AIC Model likelihoods (L) and weights (W) Model 1: L = 0.872538, W = 0.465965 Model 2: L = 1, W = 0.534035

This result implies that the more complex model is *ℓ*_21_ = 1/0.872538 = 1.15 times more likely than the simpler mode, which can be reported as follows: *a model that assumes that participants experience different anxiety on average to real spiders than to pictures of spiders is* *ℓ*_21_ = 1.15 *times more likely than a model in which participants on average experience the same level of anxiety; such a likelihood ratio can be characterised as “barely worth mentioning”, so that both models can be considered to be approximately equivalent.*

**Fig. 3:**
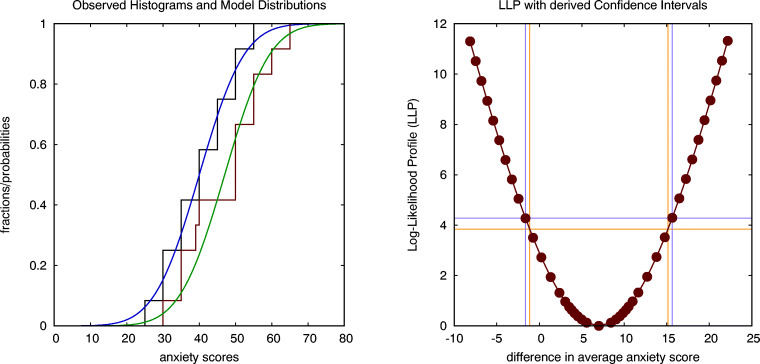
The stepwise curves in the left figure show the observed histograms for the anxiety scores, while the continuous curves are the best fitting Gaussian distributions (with equal variance) that are used to model them. The figure on the right shows the log-likelihood profile (LLP) for the difference in average scores, intersected at two levels (corresponding to a ${\chi _{1}^{2}}$ criterion and an *F*_1,23_ criterion) to establish two distinct but similar estimates for the confidence interval (CI) of the difference in averages (spider data: parametric across-subject analysis)

We can adopt the model with three parameters, two averages and one (shared) standard deviation, to create two distributions that closely fit to the observed histograms in both conditions, as shown in the left graph of Fig. 3. Optimising the lack-of-fit criterion LLC results in the expected estimates for the averages in both conditions (40 and 47 in the conditions where a picture or a real spider is used, respectively), and an estimate for the standard deviation *σ* = 9.764 that deviates slightly from the value of the standard deviation *sd* = 10.198 reported in the independent *t* test. This translates into values for Cohen’s *d* = 0.717, *JND* = 0.507 and a probability of superiority of *P*_*s*_= 0.694 that also deviate slightly from those reported by the *t* test.

This model with three parameters can be used to calculate the LLP for the difference between the two averages, and to derive a CI from this LLP based on Wilks’ theorem, as shown in the right graph of Fig. 3. More specifically, intersecting the LLP for the difference between the two averages at the chi-squared value with one degree of freedom, i.e., ${\chi _{1}^{2}}(1-\alpha =0.95)=3.84$, results in a 95% confidence interval equal to CI(dif)=[-1.13,15.13] for the difference in average anxiety scores, which is slightly smaller than the CI(dif)=[-1.63,15.63] found earlier using the independent *t* test (which serves as a reference as it is based on an exact analytical expression). An alternative is to intersect the LLP at the value of the F-distribution with (1,*N* − 1) degrees of freedom, i.e., *F*_1,23_(1 − *α* = 0.95) = 4.28, which results in an estimate for the confidence interval CI(dif)=[-1.62,15.62] of the difference in average anxiety scores that is almost identical to the CI found using the *t* test.

**Fig. 4:**
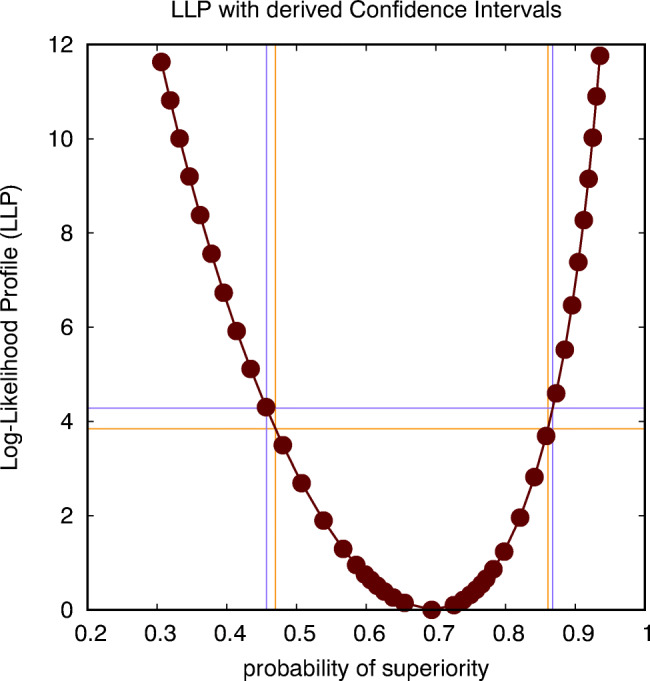
This figure shows the log-likelihood profile (LLP) for the probability of superiority, intersected at two levels (corresponding to a ${\chi _{1}^{2}}$ criterion and an *F*_1,23_ criterion) to establish two distinct but similar estimates for the confidence interval (CI) of this probability (spider data: parametric across-subject analysis)

An important advantage of Wilks’ theorem is that it also allows for estimating CIs for measures of effect size, such as Cohen’s *d* or the probability of superiority *P*_*s*_. In Fig. 4, we show the LLP for the probability of superiority, resulting in a CI(*P*_*s*_)=[0.470,0.861] in case the intersection is performed at a level of 3.84, and CI(*P*_*s*_)=[0.457,0.868] in case the intersection is performed at a level of 4.28. These estimates can be used to complete the report on the statistical analysis that we proposed earlier, i.e., that *there is an estimated probability of* *P*_*s*_= 0.694, *with a 95% confidence interval equal to CI*(*P*_*s*_)=[0.470,0.861], *according to a *${\chi _{1}^{2}}$ *criterion, or CI*(*P*_*s*_)=[0.457,0.868], according to an *F*_1,23_ criterion, for participants to report a higher anxiety score when confronted with a real spider instead of with the picture of a spider; a probability at chance level *P*_*s*_ = 0.5 *can hence not be excluded with 95% confidence*. According to Table 2, the effect size is hence in between non-existing and 1 JND, which is quite a large range because the number of observations is rather limited.

### Receiver operating characteristic (ROC)

An alternative way to characterise the difference between two experimental conditions, borrowed from communication theory, is by means of the Receiver Operating Characteristic (ROC). In empirical research we create two conditions and estimate the distribution of the dependent variable in both of them. Communication theory addresses the reverse problem, given an observed value of the dependent value, can we derive which condition it was generated in. This is accomplished by selecting a threshold value and choosing one condition if the observed value is lower than this threshold, and the other condition if the observed value is higher than this threshold. It results in two types of errors (called types I and II in statistics), i.e., choosing condition 2 when the value was generated in condition 1, and choosing condition 1 when the value was generated in condition 2. Once the cost of each type of error is known, the threshold value can be chosen to minimise the overall cost.

**Fig. 5:**
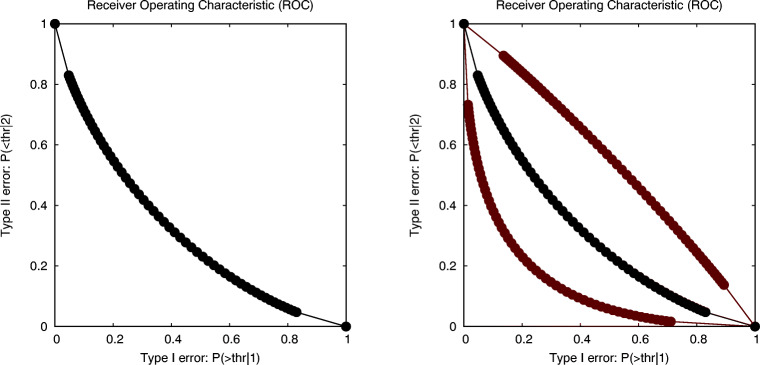
The receiver operating characteristic (ROC) in the left figure shows the relationship between the probabilities of both types of errors when an observed value is used to decide the condition that it was generated in. Each *dot* on the curve corresponds to a different value for the decision threshold. The figure on the right shows two additional curves that are the upper and lower boundaries for the 95% confidence area for this ROC (spider data: parametric across-subject analysis)

However, as such costs are often not provided, an alternative is to create the ROC, where each point on the curve shows the values for the two types of errors and corresponds to a different threshold value for deciding between both conditions. In case the two experimental conditions are modelled by parametric distributions, such a curve can be easily generated. The result for our (across-subject) experiment, assuming Gaussian distributions with equal variance in both conditions, is shown in Fig. 5.

The link to our earlier discussion is that it has been proven that the probability of superiority is equal to *P*_*s*_ = 1 − *AUC*, where *AUC* is the area under the ROC curve. While generating the ROC corresponding to two estimated distributions in distinct conditions is fairly trivial, modern statistics also allows to derive an (asymptotically) correct confidence area for this ROC. In the right graph of Fig. 5, we show the two ROC curves that bound the 95% confidence area. The areas under these two extreme ROC curves correspond to the lower and upper bounds for the 95% CI for the probability of superiority.

## Interactive statistics with ILLMO

In this section, we introduce the interface of the interactive statistical program ILLMO (Martens, 2012; 2014; 2017), and we show how the different analyses discussed in the previous section can be activated from within this interface. We only present a brief introduction with an explanation of the major features that are relevant for the current paper, and we refer to earlier publications (Martens, 2012; 2014) and the website for the ILLMO project (http://illmoproject.wordpress.com) for more extensive information, including detailed descriptions and instruction videos on a diverse range of statistical analyses that can be performed with the program.

**Fig. 6:**
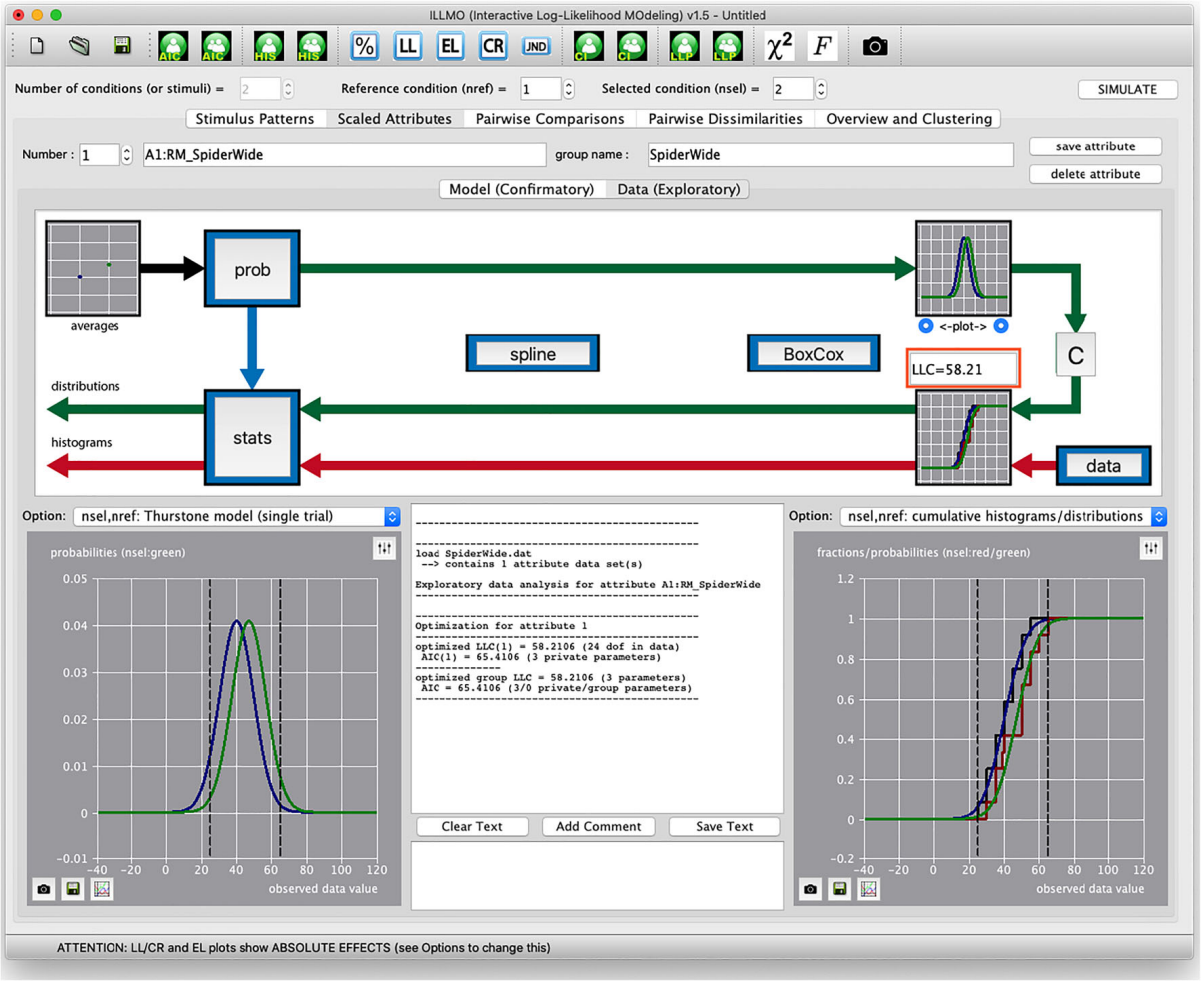
The interface of the ILLMO program after entering the data from the “spider” experiment

In Fig. 6, we show the interface after the “spider” data has been loaded. The interface provides access to both the within-subject analysis, under “pairwise comparisons”, and the across-subject analysis, under “scaled attributes”, but only the latter part is visible in the figure. The number of conditions has been set to two in the upper left corner. The observed histogram and Gaussian model distribution for the reference condition (1) are shown in black and blue, respectively, while the histogram and distribution for the selected condition (2) are shown in red and green, respectively. The minimised value for the lack-of-fit criterion LLC is displayed in the orange box. The upper diagram illustrates the main process that occurs within the ILLMO program: the observed histograms that constitute the inputs being provided are represented by the red arrow at the bottom of the diagram, and are approximated by (parametric) distributions that are represented by the green arrow at the top of the diagram. The lack-of-fit criterion LLC is minimised to establish the optimal values for the distribution parameters. The two plot windows in the lower part of the figure offer a large number of options (over 50 in total) to visualise different aspects of the statistical analysis, while the window in between is used for textual output. The small textual window below it allows the user to insert textual comments on the reported results of the analyses. The textual output can be cleared or saved to a file at any time.

**Fig. 7:**
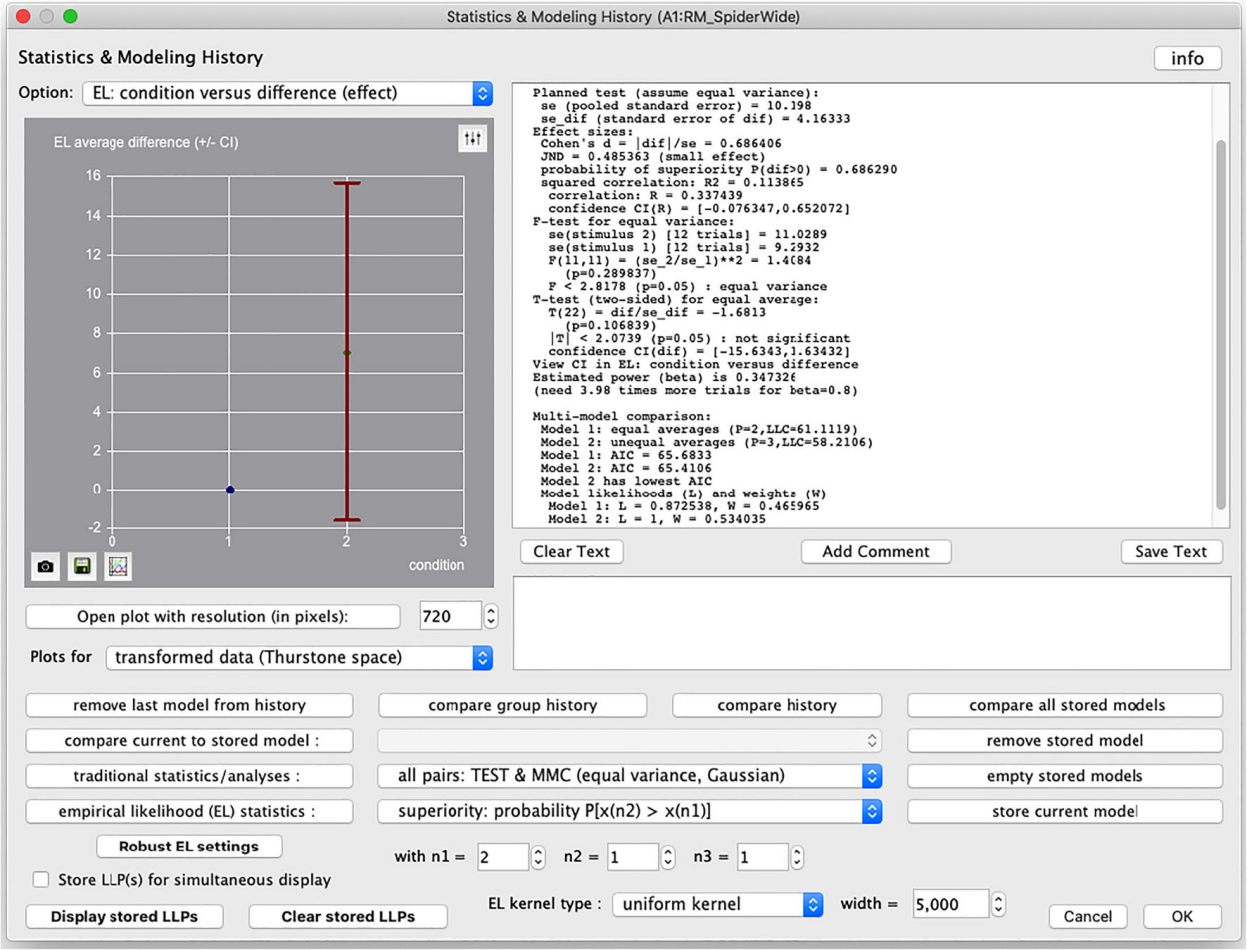
The “stats” dialog window of the ILLMO program that, amongst others, provides access to a wide range of traditional statistical methods. In the example being shown, an across-subject *t* test, followed by a multi-model comparison, has just been executed and the result is reported in both textual and graphical form

There are two buttons, labelled “prob” and “stats” that provide access to the analyses that we have discussed in the previous section. More specifically, the “stats” button opens a dialog box that provides, amongst others, access to traditional statistical methods. In Fig. 7 we have selected the option “all pairs: TEST & MMC (equal variance, Gaussian)” and have pressed the button “traditional statistics/analyses:” to execute two analyses in succession: the independent *t* test (TEST) and the corresponding multi-model comparison (MMC). The results are reported textually in the window in the upper-right corner, and graphically in the window in the upper-left corner, and correspond to the discussion on the across-subject analysis in the previous section.

**Fig. 8:**
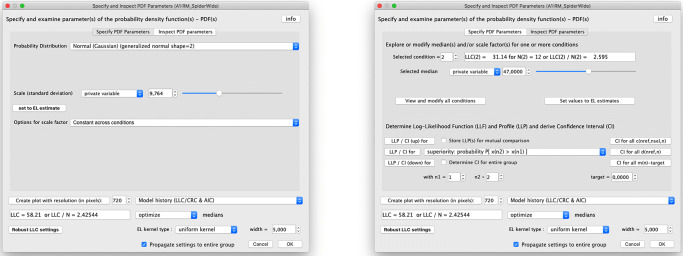
The “prob” dialog window of the ILLMO program contains two pages. The first page, on the left, is used to specify the class of parametric distributions that is used to model the observed histograms. The second page, on the right, is used to trigger the creation of the LLP and the derivation of the CI for a selected model parameter

The “prob” button opens a dialog window with two pages, as shown in Fig. 8. The first page is shown on the left and allows, amongst others, to choose the family of parametric distributions that is to be used to model the observed histograms. There are approximately ten different two-sided distributions to choose from, including the logistic and Student’s *t* distribution, and 20 different one-sided distributions, including the Chi-squared, log-normal and Poisson distribution. The default choice is the Gaussian (or normal) distribution, as this is the choice adopted by many traditional statistical methods (such as *t* tests, ANOVA, linear regression, etc.). Another option that can be specified on this page is whether of not the scale factor, which is equal to the standard deviation in case of Gaussian distributions, should be the same for all conditions or vary across conditions. The second page, shown on the right in Fig. 8, is used to trigger the calculation of a LLP, and the subsequent derivation of the CI using Wilks’ theorem, for a selected model parameter. In the figure, the selection is set to “superiority: probability *P*[*x*(*n*2) > *x*(*n*1)]”, which corresponds to the probability of superiority discussed in the previous section, and the analysis is started by pressing the button “LLP/CI for” in front of it. The ROC of Fig. 5 that is created in case this selection is made appears in a separate window once the calculation of the CI has finished. Other model parameters such as the average for a single condition, the difference between the averages in two distinct conditions, the shared scale factor, the effect size (such as Cohen’s *d*) between two distinct conditions, etc. can be selected as alternatives.

A dialog box that allows to set the required level of confidence, which is equal to 95% by default, will appear upon pressing the button labelled “%” in the main interface of Fig. 6. For instance, when constructing a 95% CI for a one-sided test instead of a two-sided test, this confidence level should be lowered to 90%. The “Options” menu allows to switch between the default option of using the ${\chi _{1}^{2}}(1-\alpha )$ level to intersect the LLP when deriving a CI and the alternative option of using the *F*_1,*N*− 1_(1 − *α*) level, which is likely to produce a better approximation when the number of observations *N* is small.

## Non-parametric statistics

As indicated before, in cases where the observed histograms cannot be approximated by Gaussian distributions, methods such as *t* tests are not advised. Typical examples are for instance: 1) when histograms are not uni-modal, for instance indicating the presence of two or more subgroups of subjects with distinct behaviours, or 2) when histograms are heavily skewed to one side, for instance when the dependent variable is strictly positive and there are large differences between subjects (e.g., in performance time) (Huff, 1954). The traditional advice in such cases is to use non-parametric tests. These tests come in the same two flavours as the parametric tests. When analysing within-subject data, the advice is to use the Wilcoxon signed-rank test. When analysing across-subject data, the advice is to use the Wilcoxon rank-sum test (or the closely related Mann–Whitney test). As before, we will start from the reports typically generated by these methods before proposing alternatives.

In chapter 15 of the book “Discovering Statistics using SPS”, entitled “Non-parametric Tests”, Andy Field introduces a simple data set that is reproduced in Table 4. The participants in the experiment either took ecstasy or alcohol on Saturday evening, and were asked to fill in the Beck Depression Inventory (BDI) questionnaire two times in the days to follow: once on Sunday and once on Wednesday. The responses from one questionnaire were used to generate a single dependent variable, i.e., the BDI scores shown in the table.

**Table 4:** BDI scores on two different days (Sunday and Wednesday) in response to different drug use (ecstasy or alcohol) on Saturday evening (from Field, 2013)

Participant	Drug	Sunday	Wednesday	Difference
1	Ecstasy	15	28	13
2	Ecstasy	35	35	0
3	Ecstasy	16	35	19
4	Ecstasy	18	24	6
5	Ecstasy	19	39	20
6	Ecstasy	17	32	15
7	Ecstasy	27	27	0
8	Ecstasy	16	29	13
9	Ecstasy	13	36	23
10	Ecstasy	20	35	15
11	Alcohol	16	5	-11
12	Alcohol	15	6	-9
13	Alcohol	20	30	10
14	Alcohol	15	8	-7
15	Alcohol	16	9	-7
16	Alcohol	13	7	-6
17	Alcohol	14	6	-8
18	Alcohol	19	17	-2
19	Alcohol	18	3	-15
20	Alcohol	18	10	-8

### Within-subject analysis

For a within-subject analysis, we use the differences in the BDI scores for the same subject on two different days, shown in the last column of Table 4, so that the day of the week is considered to be the independent variable. We will use the data from the *N* = 10 subjects who took alcohol, as the deviation from a normal distribution of these difference scores was substantially larger for the subjects who had taken alcohol (low probability *p* = 0.0046 of being normally distributed, according to the K-squared test for normality) than for the subjects who had taken ecstasy (high probability *p* = 0.629 of being normally distributed). The following way of reporting the outcome of the Wilcoxon signed-rank test was proposed by Field: *For alcohol users, the depression levels were significantly lower on Wednesday (median= 7.50) than on Sunday (median= 16), with test statistic* *T* = 8 *and z-score* *z* = − 1.99, *p* < 0.05, *corresponding to an effect size of* *R* = − 0.44.

The Wilcoxon test is based on the assumption that the test statistic *T*, which is derived from the rank orders of the observed differences, is (approximately) normally distributed with a known average and standard error, so that the Z-test can be applied on these rank-ordered scores. This is an untested assumption that we would like to avoid, if possible. The major issue with the Wilcoxon test is however the same as with the dependent *t* test, i.e., that an effect size (of *R* = − 0.44) is reported without any indication of the accuracy with which this effect size is determined.

It turns out that there is a recent statistical theory called empirical likelihood (EL) (Owen, 2001) that provides an elegant solution for how to handle data that cannot be readily approximated by a known parametric distribution. The approach taken in EL is to optimise over all possible distributions on the observed (difference) scores *x*_*i*_, while imposing some boundary conditions. This is accomplished by assigning a separate model parameter to each observed difference. This parameter *p*_*i*_ is the model probability that the value *x*_*i*_ occurs. Not all possible combinations of model probabilities are however allowed. The first condition is obvious, i.e., that the probabilities have to be positive and sum to one, i.e., ${\sum }_{i=1}^{N} p_{i}=1$ (otherwise, they could simply not be interpreted as probabilities).

Suppose that we now want to determine what the likelihood is that the average (difference) is equal to *μ*, then we add an extra condition that guarantees this, i.e.,

7 $$ \sum\limits_{i=1}^{N} p_{i}\cdot x_{i} = \mu, \text{or} \sum\limits_{i=1}^{N} p_{i}\cdot w(x_{i},\mu) = 0, \text{with}\ w(x_{i},\mu) =x_{i}-\mu. $$

The theory of EL has established that the optimal model probabilities in case the average (difference) is enforced to be equal to *μ* are equal to

8 $$ p_{i}(\mu) = \frac{1}{N\cdot (1 + \lambda_{m}(\mu)\cdot w(x_{i},\mu) )}, $$

for *i* = 1…,*N*, where *λ*_*m*_(*μ*) denotes the value that minimises the empirical likelihood criterion (ELC), i.e.,

9 $$ ELP(\mu) = \min\limits_{\lambda} ELC(\lambda) = \min\limits_{\lambda} 2 \sum\limits_{i=1}^{N} \log(1 + \lambda\cdot w(x_{i},\mu) ). $$

Note that the optimisation only needs to be performed over the single parameter *λ*, which is obviously a lot easier than optimising over all individual probabilities *p*_*i*_, for *i* = 1,…,*N*. It is worthwhile to remark that, by definition, *ELP*(*μ*) = 0, and *λ*_*m*_(*μ*) = 0, in case *μ* is equal to the observed average (difference) (of *μ* = − 6.3 in the experiment). For values *μ* unequal to the observed average (difference), *ELP*(*μ*) ≥ 0.

Don’t worry if the above (mathematical) explanation of EL is not completely clear, the important thing to remember is the following. While the construction of the ELP may seem complicated, partly because it involves numerical optimisation (to find the optimal value for *λ*), the outcome is a measure *ELP*(*μ*) that expresses the lack-of-fit between the observed histogram of differences in BDI scores and the *best fitting distribution on the observed (difference) values* that satisfies the condition that the average (difference) is equal to *μ*. This is similar to the LLP in parametric statistics that expresses the increase in lack-of-fit between the observed histogram and the best fitting distribution from a parametric class of distributions (such as Gaussian distributions) where one of the distribution parameters (such as the average) is fixed to a non-optimal value. Note that we also didn’t explain in much (mathematical) detail how and why the LLC and LLP work; understanding the interpretation is sufficient for most applications.

The ELP can be used in a similar way as the LLP to create confidence intervals. The construction of an ELP can be triggered in ILLMO using the button labelled “empirical likelihood (EL) statistics:” in the “stats” window, as shown in Fig. 7. The resulting ELP has been shown to also satisfy Wilks’ theorem (Owen, 2001), which means that an (asymptotically correct) approximation to the CI for the average can be found by intersecting the ELP at a level that is dictated by the required level of confidence. As in the case of parametric statistics, we will report two CIs for our example experiment, i.e. *CI*(*dif*) = [− 9.57,− 1.22] is obtained when intersecting the ELP at the level ${\chi _{1}^{2}}(1-\alpha =0.95)=3.84$, while CI(dif)=[-10.03,-0.30] when intersecting the ELP at the level *F*_1,9_(1 − *α* = 0.95) = 5.12. As zero is outside the estimated CIs we can conclude that: *for alcohol users, the depression levels were on average* *dif* = − 6.3 *lower on the BDI scale on Wednesday than on Sunday, which is, according to an empirical likelihood analysis, (marginally) significant at 95% confidence as both the CI according to the *${\chi _{1}^{2}}$ *criterion* *CI*(*dif*) = [− 9.57,− 1.22] *and the CI according to the* *F*_1,9_ *criterion CI(dif)=[-10.03,-0.30] exclude zero*.

**Fig. 9:**
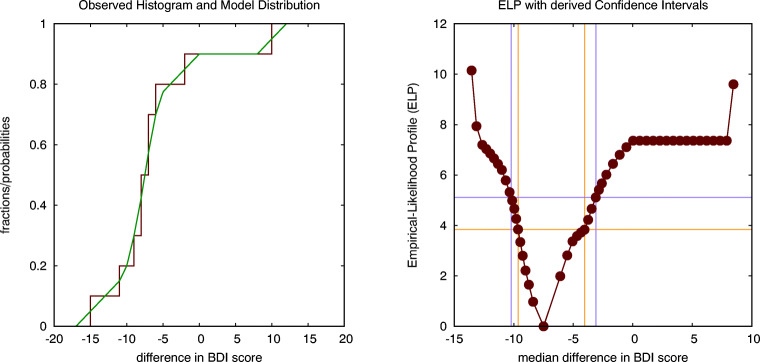
The stepwise curve in the left figure shows the observed histogram for the differences in BDI scores, while the continuous curve is obtained after smoothing with a uniform kernel function of width equal to 4 (units of BDI). The figure on the right shows the empirical-likelihood profile (ELP) for the median difference, intersected at two levels (corresponding to a ${\chi _{1}^{2}}$ criterion and an *F*_1,9_ criterion) to establish two distinct but similar estimates for the confidence interval (CI) of the median difference (alcohol data on Sunday and Wednesday: non-parametric within-subject analysis)

It is well known that the average is not a robust statistic, in the sense that its value can be heavily influenced by outliers, which is why such outlier values are often removed before applying parametric statistics, especially if such statistics are based on the use of Gaussian distributions. A more robust statistic in this sense is the median (difference) value, which is equal to *m* = − 7.5 in our example case. One advantage of the Wilcoxon signed-rank test is that it is based on the rank-order of the observed values, and hence has this robustness built into it. The EL method can however also be applied to median values rather than to average values by making only a small modification to the above derivation, i.e., by adopting an alternative expression for the weight function

10 $$ w(x_{i},m) = K(m-x_{i}) - 0.5, $$

for *i* = 1,…,*N*, where *m* is the median value that the model distribution should satisfy, 0.5 is the probability that corresponds to a median value, and *K*(*x*) is a so-called (integrated) kernel function that is centred on zero and that changes continuously from 0 to 1. The effect of introducing this kernel function (Chen & Hall, 1993) is that we approximate the observed histogram, which is a stepwise-function, by a continuous function, as shown in the left graph in Fig. 9. Such a continuous function guarantees that we the median is a uniquely defined value. This method of visualising histograms is well-known as it is also used to create so-called kernel density functions, i.e., a kernel function is used to smooth an observed histogram that is only defined for discrete values so that it resembles a continuous function. In case of Fig. 9, we have used a uniform kernel of width equal to 4 (units of BDI), so that the stepwise transitions in the observed histogram are replaced by linear transitions (of width 4).

The ELP for the median is shown in the right graph of Fig. 9. More specifically, intersecting the ELP for the median difference at the Chi-squared value with one degree of freedom, i.e., ${\chi _{1}^{2}}(0.95)=3.84$, results in a 95% confidence interval of CI(dif)=[-9.64,-4.05] for the median difference in BDI scores, while intersecting the LLP at the value of the F-distribution with (1,*N* − 1) degrees of freedom, i.e., *F*_1,9_(0.95) = 5.12, results in a confidence interval of CI(dif)=[-10.22,-3.10]. Both confidence intervals do not include zero, so that the earlier conclusion, based on using the average (difference), that there is a significant decrease in BDI scores from Sunday to Wednesday is not affected by this switch to median (difference). Note that the effect on the median is more pronounced than the effect on the average, as the zero value is further removed from the (upper) boundary of the CI.

While the exact choice of the kernel function *K*(*x*) being used might seem to be very important, practice shows that the estimated CIs are very robust to moderate changes in this kernel function. The interface of the ILLMO program allows the user to experiment with kernel functions of different shapes and widths and to visually inspect the relationship between the observed histogram and the interpolated distribution, as well as the impact on the estimated CI.

**Fig. 10:**
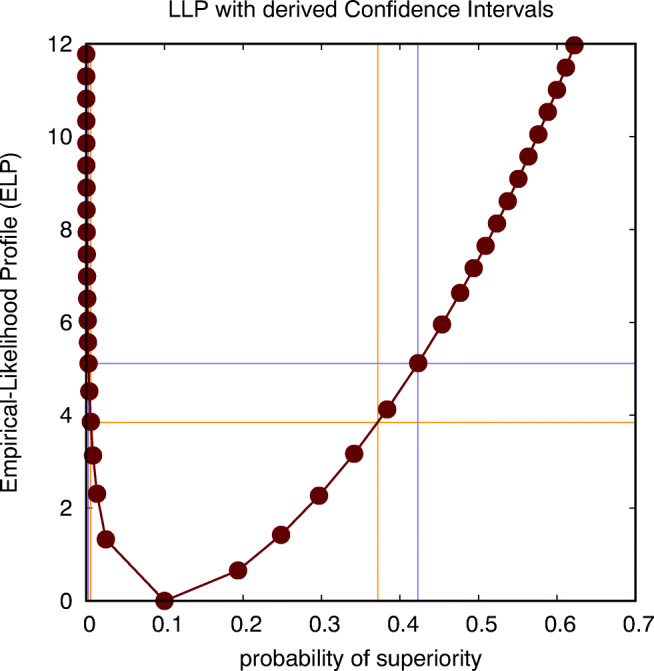
This figure shows the empirical-likelihood profile (ELP) for the probability of superiority, intersected at two levels (corresponding to a ${\chi _{1}^{2}}$ criterion and an *F*_1,9_ criterion) to establish two distinct but similar estimates for the confidence interval (CI) of this probability (alcohol data on Sunday and Wednesday: non-parametric within-subject analysis)

A major advantage of the theory of EL is that it can be extended to other model parameters then the average and median, such as the probability of superiority (i.e., the model probability that a value larger than zero is observed), i.e., by simply changing the weight function to

11 $$ w(x_{i},P_{s}) = K(-x_{i}) - (1-P_{s}), $$

for *i* = 1,…,*N*, where *P*_*s*_ is the probability of superiority that the model distribution should satisfy. Similarly as in the case of the median, a smoothing kernel *K*(*x*) is required. The probability of superiority is *P*_*s*_ = 0.1 and agrees with the probability of 1 − *P*_*s*_ = 0.9 for the smoothed histogram in Fig. 9 when the difference in BDI score is equal to zero. In Fig. 10, we show the ELP for the probability of superiority, resulting in a CI(*P*_*s*_)=[0.006,0.372] in case the intersection is performed at a level of 3.84, and CI(*P*_*s*_)=[0.003,0.423] in case the intersection is performed at a level of 5.12. These estimates can be used to formulate the following report on the statistical analysis, i.e., that *there is an estimated probability of* *P*_*s*_= 0.1, with a 95% *confidence interval equal to CI*(*P*_*s*_)=[0.006,0.372], *according to a *${\chi _{1}^{2}}$ *criterion, or CI*(*P*_*s*_)=[0.003,0.423], *according to an* *F*_1,9_ *criterion, for participants to report a higher BDI score on Wednesday than on Sunday, according to an analysis using empirical likelihood with a uniform smoothing kernel of width 4 (units of BDI)*. Note that significance corresponds to the fact that the chance level (*P*_*s*_ = 0.5) is outside of these estimated CIs. According to Table 2 the effect size is hence in between small and 2.5 JND, which is quite a large range because the number of observations is rather limited.

### Across-subject analysis

For an across-subject analysis, we will use the responses on one of the two days that BDI scores were collected, so that the drug taken is considered to be the independent variable. We will, somewhat arbitrarily, use the data from the *N* = 20 subjects on Sunday (on Sunday, the BDI scores for ecstasy had a low probability *p* = 0.015 of being normally distributed, according to the K-squared test for normality, while there was no such evidence for the BDI scores for alcohol, who had a high probability *p* = 0.706 of being normally distributed; on Wednesday, the BDI scores for alcohol had a low probability *p* = 0.0014 of being normally distributed, while there was no such evidence for the BDI scores for ecstasy with a high probability *p* = 0.672 of being normally distributed). The following way of reporting the outcome of the Wilcoxon rank-sum test was proposed by Field: *Depression levels in ecstasy users (median= 17.5) did not significantly differ from alcohol users (median= 16) the day after the drugs were taken, with test statistic* *W*_*s*_ = 90.5 *and z-score* *z* = − 1.11, *p* > 0.05, *and an effect size of* *R* = − 0.25.

**Fig. 11:**
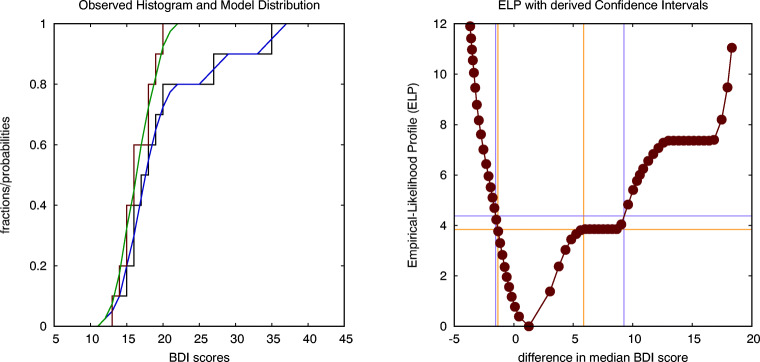
The stepwise curves in the left figure shows the observed histograms (*black*: ecstasy, *red*: alcohol) for the BDI scores on Sunday, while the continuous curves (*blue*: ecstasy, *green*: alcohol) are obtained by smoothing with a uniform kernel function of width equal to 4 (units of BDI). The figure on the right shows the empirical-likelihood profile (ELP) for the difference in medians, intersected at two levels (corresponding to a ${\chi _{1}^{2}}$ criterion and an *F*_1,19_ criterion) to establish two distinct estimates for the confidence interval (CI) of the difference in medians

Similarly as in the case of non-parametric within-subject analysis, we can use the ELP to estimate the CI for the difference in the median values. In order to do so, we again need to use a smoothing kernel. In the left graph of Fig. 11 we show both the original histograms as stepwise curves and the smoothed histograms as piecewise linear curves. The ELP for the difference in medians is shown in the right graph of Fig. 11. More specifically, intersecting the ELP for the difference in medians at the chi-squared value with one degree of freedom, i.e., ${\chi _{1}^{2}}(0.95)=3.84$, results in a 95% confidence interval equal to CI(dif)=[-1.34,5.88] for the difference in median BDI scores, while intersecting the LLP at the value of the F-distribution with (1,*N* − 1) degrees of freedom, i.e., *F*_1,19_(0.95) = 4.38, results in a confidence interval equal to CI(dif)=[-1.53,9.28]. Both confidence intervals include zero, which is in agreement with the conclusion from the Wilcoxon rank-sum test of a non-significant difference in the medians between both conditions.

**Fig. 12:**
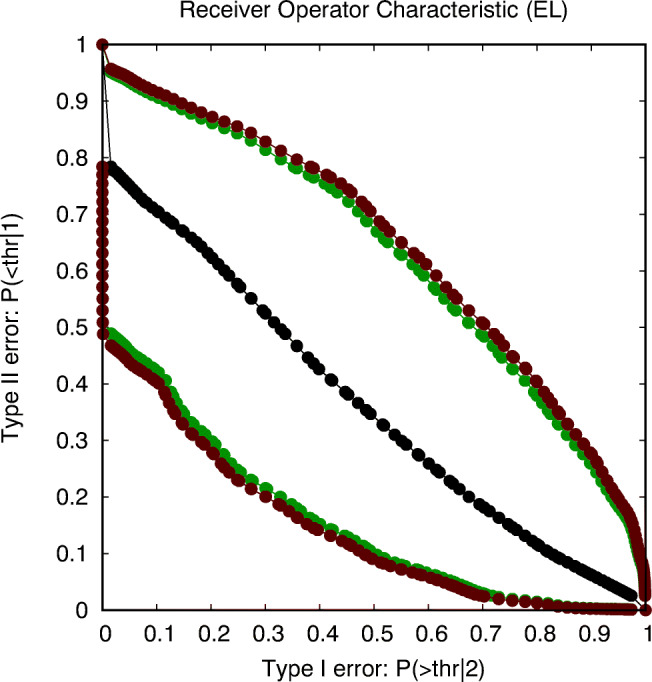
The graph shows the ROC (*in black*) together with its 95% confidence region as determined using empirical likelihood; the green boundaries correspond to a ${\chi _{1}^{2}}$ criterion, while the red boundaries correspond to an *F*_1,19_ criterion (drug data on Sunday: non-parametric across-subject analysis)

As explained before, we are more interested in estimating effect size than in establishing significance. We follow a method proposed in (Claeskens, Jing, Peng, & Zhou, 2003), which consists of first constructing the 95% confidence area for the ROC, the result of which is shown in Fig. 12. It is worthwhile to note that we are not aware of any other readily available program that currently implements this method. The area *AUC* under the ROC can be used to estimate the probability of superiority *P*_*s*_ = 1 − *AUC* = 0.634, as well as CI(*P*_*s*_)=[0.375,0.846], in case the intersection is performed at a level of ${\chi _{1}^{2}}=3.84$ (green curves), and CI(*P*_*s*_)=[0.357,0.857] in case the intersection is performed at a level of *F*_1,19_ = 4.38 (red curves). These estimates allow us to formulate the following report on the statistical analysis, i.e., that *there is an estimated probability of* *P*_*s*_= 0.634, *with a 95% confidence interval equal to CI*(*P*_*s*_)=[0.375,0.846], *according to a *${\chi _{1}^{2}}$ *criterion, or CI*(*P*_*s*_)=[0.357,0.857], *according to an* *F*_1,19_ *criterion, for participants to report a higher BDI score on Sunday for ecstasy than for alcohol, according to an analysis using empirical likelihood with a uniform smoothing kernel of width 4 (units of BDI)*. Note that this is not significant as the chance level (*P*_*s*_ = 0.5) is inside of these estimated CIs.

## Analysing discrete (ordinal) data

In a CHI paper from 2010 (Kaptein & Robertson, 2012), Kaptein et al. argue that evaluations of subjective experiences within the CHI community make extensive use of Likert scales, but that the statistical methods used for processing such data, such as *t* tests or ANOVA, are not intended for analysing discrete ordinal data, but instead, for analysing continuous interval data. One of the reasons mentioned for this sub-optimal practice is the absence of software that supports alternative types of analyses and that is readily available for HCI scientists. In a follow-up book (Robertson & Kaptein, 2016) entitled “Modern Statistical Methods for HCI” the authors promote the use of the R language as a way for HCI scientists to gain access to modern statistical methods. R is a platform that is supported by a large community and probably the most likely venue for new statistical methods to be made available by their developers. While R is extremely powerful and useful for statisticians and data scientists who use it on a regular basis, it is still a programming language with a non-trivial syntax that requires time and effort to learn and master. The fact that it is a programming language also means that programming mistakes will inevitably be made, and that substantial time may be lost to debug and correct such mistakes, or that such mistakes may simply remain unnoticed. It therefore seems unlikely that many HCI scientists, or psychologists, who probably view statistics more as something that they use rather than as a core competency that they need to develop in more depth, will be tempted to make such an investment. The current paper promotes the ILLMO program as an alternative, more user-friendly, platform for gaining access to modern statistical methods. Content-wise, this paper supports the view of the authors of the above book that there is an urgent need to adopt more modern statistical methods in the HCI practice. This paper also contributes additional methods to the ones already discussed in the aforementioned book, such as constructing confidence intervals for effect sizes, using empirical likelihood as an alternative for non-parametric tests, etc.

In the CHI paper from 2010, the focus was still on analysing significance, but in a follow-up paper (Robertson & Kaptein, 2016) from CHI 2012, Kaptein and Robertson set out to *“promote consideration of the magnitude and actual importance of effects, as opposed to statistical significance, as the new criteria for evaluating CHI research”*, a position that is in line with APA guidelines (Cumming et al., 2012) and that we wholeheartedly subscribe to in this paper. They, however, only concentrate on reporting effect sizes, not on the need to also establish the accuracy (or confidence intervals) for them. They also didn’t make a concrete proposal for how to address the discrete nature of the ordinal data produced by Likert scales, as they continued to rely on statistical outcomes, such as Cohen’s d, produced by traditional methods such as *t* tests.

We will use the same dataset that was used in the CHI 2012 paper to illustrate our concrete proposal for how to take the discrete nature of the data into account in a statistical analyses. This data is available as a file entitled “data40.csv” on the ILLMO website. It contains (simulated) data on comparing the ease of use of a Windows VISTA (1) and a Mac OS-X (2) operating system by both novice and experienced users. In the data file, the column named “Between” specifies the system being tested (1 or 2), which we designate as the independent variable. The column named “Score” contains the (simulated) outcomes on a Likert scale in a usability questionnaire and is hence equal to the dependent variable. The higher the number, the more the participant appreciated the ease-of-use of the operating system. The column named “Time” specifies whether the measurement was performed with novices (1) or experienced users (2), but in the current example we will only use the data from the novice users. There are *N*_1_ = 22 measurements in condition 1, *N*_2_ = 18 measurements in condition 2, or *N* = 40 measurements in total.

The (discrete data) from the experiment is summarised in Table 5. The columns with the integer labels contain the number of times that a response in one of the integer categories 1 to 7 was produced in each of the two conditions. These counts for each of the two conditions can be converted into fractions by dividing by the total number of responses in a condition. A statistical model will generate probabilities for the same categories, and the best model is obviously the one where there is a close fit, according to a suitable lack-of-fit criterion such as log-likelihood, between the observed fractions and the model probabilities.

**Table 5:** Counts per (discrete) category for each of the two conditions in the simulated usability experiment (from Kaptein & Robertson, 2012).

Condition	1	2	3	4	5	6	7	Total
VISTA	2	1	2	11	5	1	0	22
OS-X	2	1	1	2	5	5	2	18

ILLMO uses *Thurstone modeling* to model the probabilities in the ordinal categories 1–7. This approach is named after a notorious researcher in the 1920s who wondered how continuous distribution functions such as the Gaussian distribution function could be used to generate probabilities for discrete data, and who came up with the following elegant proposition (Thurstone, 1927; Torgerson, 1958; Boschman, 2000). He observed that the integer categories lie in between boundaries at half-integer values, and he proposed to use the area under a continuous distribution function between two such boundaries as the model prediction for the probability for the corresponding interval. An exception is made for the first and last category where the lower or upper boundary are equal to minus and plus infinity, respectively. In this way, the parameters of the continuous distribution function, such as the average and standard deviation of a Gaussian function, indirectly determine the probabilities for all categories.

An advantage of Thurstone modelling is that the intuitive interpretation of continuous distributions remains intact and only the log-likelihood criterion (LLC) needs to be adjusted to reflect the discrete nature of the data, i.e.,

12 $$ LLC_{i} = \sum\limits_{j=1}^{7} n_{ij}\cdot 2 \log\left( \frac{n_{ij} }{N_{i}\cdot p_{ij}} \right), $$

where {*n*_*ij*_, for *j* = 1,…,7} are the observed counts in condition *i*, for *i* = 1,2 (see Table 5), and {*p*_*ij*_, for *j* = 1,…,7} are the corresponding probabilities predicted by the Thurstone models. Note that each condition is modelled by a different underlying continuous distribution function. This LLC has been shown to satisfy all the properties that we relied on when discussing modern statistical methods for continuous data. For one, the LLC for a complete experiment including two conditions is obtained by adding the LLC values for each of these conditions. This total *LLC* = *LLC*_1_ + *LLC*_2_ can be combined with the total number of parameters *P* (across all conditions) to define the AIC in exactly the same way as before.

For instance, a first model that corresponds to a null hypothesis *H*_0_ would contain *P* = 2 parameters, corresponding to using the same Gaussian distribution for both conditions, while a second model that corresponds to the alternative hypothesis *H*_1_ would contain *P* = 3 parameters, corresponding to each condition using a Gaussian distribution with a different average value, but adopting the same standard deviation for both distributions. We can determine the LLC and AIC for both models being applied to our example data, i.e., *LLC* = 20.63 and *AIC* = 25.97 for the first model that corresponds to the null hypothesis *H*_0_, and *LLC* = 18.01 and *AIC* = 27.01 for the second model that corresponds to the alternative hypothesis *H*_1_. The result can be reported as follows: *a multi-model comparison between two models, one assuming an equal average value for the usability score in both conditions and one assuming unequal averages, results in a likelihood ratio of* *ℓ*_12_ = 1.63 *in favour of the first (simplest) model; this ratio is “hardly worth mentioning”, according to* Table 3, *and can be interpreted as support for the null hypothesis that the condition has no effect on the observed scores.* Interestingly enough, this likelihood ratio is very close to the likelihood ratio of 1.53 that was reported in (Kaptein & Robertson, 2012) based on a completely different statistical approach, i.e., a Bayesian *t* test.

We can also use the above LLC for discrete data to derive LLPs for a number of model parameters, such as individual averages per condition, the (shared) standard deviation, the difference between averages and effect sizes such as Cohen’s *d* or the probability of superiority. These LLPs have been shown to satisfy Wilks’ theorem, so that intersecting such an LLP at a level dictated by the required significance produces (asymptotically correct) estimates for the confidence intervals for such parameters. We will not illustrate the outcomes for all possible model parameters, but will restrict us here to the effect size parameter that we have been promoting in this paper, i.e., the probability of superiority.

**Fig. 13:**
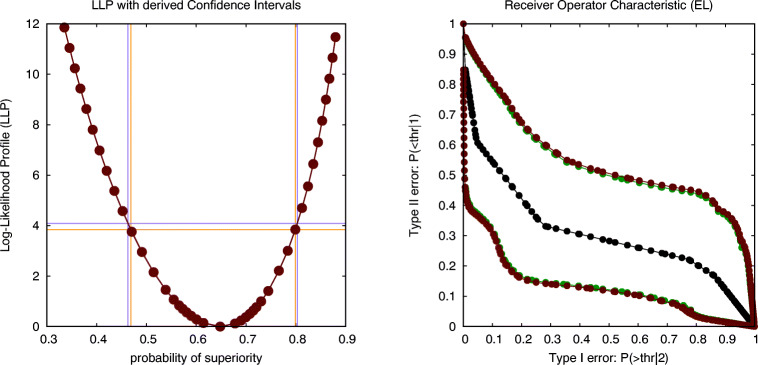
The figure on the left shows the log-likelihood profile (LLP), according to a parametric (Thurstone) model, for the probability of superiority, intersected at two levels (corresponding to a ${\chi _{1}^{2}}$ criterion and an *F*_1,39_ criterion) to establish two distinct but similar estimates for the confidence interval (CI) of this probability. The figure on the right shows the ROC (*in black*) together with its 95% confidence region as determined using empirical likelihood; the *green boundaries* correspond to a ${\chi _{1}^{2}}$ criterion, while the *red boundaries* correspond to an *F*_1,19_ criterion (usability data determined using a Likert scale)

In the left graph of Fig. 13, we show the LLP for the probability of superiority *P*_*s*_ = 0.646, resulting in a CI(*P*_*s*_)=[0.469,0.799] in case the intersection is performed at a level of ${\chi _{1}^{2}}(0.95)=3.84$, and CI(*P*_*s*_)=[0.463,0.803] in case the intersection is performed at a level of *F*_1,39_(0.95) = 4.09. These estimates allow us to formulate the following report on the statistical analysis, i.e., that *there is an estimated probability of* *P*_*s*_= 0.646, *with a 95% confidence interval equal to CI*(*P*_*s*_)=[0.469,0.799], *according to a *${\chi _{1}^{2}}$ *criterion, or CI*(*P*_*s*_)=[0.463,0.803], according to an *F*_1,39_ *criterion, for participants to report a higher usability score for the OS-X system than for the VISTA system, according to a parametric Thurstone model where the two conditions are modelled by Gaussian distributions with distinct averages and a shared standard deviation*. Note that this is a *non-significant* difference as the chance level (*P*_*s*_ = 0.5) is inside of these estimated CIs. A more precise conclusion is that *the effect is in between non-existing and large-sized* (according to Table 2).

As an alternative to the parametric Thurstone model we can also use the non-parametric method of empirical likelihood to estimate the effect size and its CI. In the right graph of Fig. 13, we show the ROC (in black) with its confidence area according to an EL estimate. The areas under these curves correspond to a probability of superiority equal to *P*_*s*_ = 0.690, with a CI(*P*_*s*_)=[0.463,0.867] in case the calculation is performed according to a ${\chi _{1}^{2}}(0.95)=3.84$ criterion, and CI(*P*_*s*_)=[0.456,0.871] in case the calculation is performed according to a *F*_1,39_(0.95) = 4.09 criterion. These estimates allow us to formulate the following report on the statistical analysis, i.e., that *there is an estimated probability of* *P*_*s*_ = 0.690, *with a 95% confidence interval equal to CI*(*P*_*s*_)=[0.463,0.867], *according to a *${\chi _{1}^{2}}$ *criterion, or CI*(*P*_*s*_)=[0.456,0.871], *according to an* *F*_1,39_ *criterion, for participants to report a higher usability score for the OS-X system than for the VISTA system, according to an analysis using empirical likelihood with a uniform smoothing kernel of width 1 (units on the usability scale).* According to this non-parametric EL analysis, this is a *non-significant* difference as the chance level (*P*_*s*_ = 0.5) is inside of these estimated CIs. A more precise conclusion is that *the effect is in between non-existing and 1 JND* (according to Table 2).

## Conclusions

In their CHI 2012 paper (Kaptein & Robertson, 2012), Kaptein and Robertson express the following recommendations for future CHI authors, which we use to identify some of the contributions of this paper and of the interactive statistical program ILLMO:

- *Bolder predictions predicting the direction and magnitude of effects would be beneficial:* we have interpreted this advice in the sense that not only the effect size should be estimated, which is already provided by several existing statistical methods, but that we should also be able to derive the confidence interval for such an effect size, as otherwise no inference can be made on whether or not a hypothesised effect size can be rejected based on the available experimental data.
- *We should enable the researcher to calculate the number of participants they require to detect an effect:* this is supported in the *t* tests executed by the ILLMO program, by offering exactly this information; when using CIs, we can use the “rule-of-thumb” that CIs will approximately shrink by a factor of 2 when the number of measurements increases by a factor of 4.
- *It can be beneficial to calculate the probability of the hypothesis given the data:* while Kaptein and Robertson argue in favour of using Bayesian analysis for this purpose, we instead adopt the method of multi-model comparison; the discussion about the pros and cons of both alternative methods is ongoing, but offering a software platform (ILLMO) that provides an easy access to multi-model comparison, i.e., without requiring the need to program (as would be required when using R, for instance), can help to perform the analyses that are needed to make this comparison. The outputs from ILLMO can for instance be compared with those from a software platform such as JASP (see http://jasp-stats.org) that implements Bayesian statistics.
- *We encourage researchers, reviewers, programme chairs and journal editors to work towards raising the standard of reporting statistical results:* we addressed this by proposing formulations for how to summarise the statistical analyses that we introduced, especially when these analyses involved unfamiliar methods.
- *It is good practice to interpret the non-standardised sizes of the estimated effects:* we adopted probability of superiority as our preferred measure for effect size, since we hold the opinion that it is easier to interpret than the more traditional alternatives such as Cohen’s *d* and the correlation coefficient *R*. The probability of superiority corresponds to a simple experiment that is easy to explain and understand, also for non-statisticians: randomly take one outcome from each of the two experimental conditions, it is the probability that the highest outcome was indeed produced in the condition that is hypothesised to produce the highest scores (on average).

This paper has shown, for the relatively simple task of comparing two experimental conditions, how the ILLMO software program provides access, in a user-friendly way, to a number of modern statistical methods that can provide essential information, especially on effect size, that is often lacking in more traditional methods. Some of the identified advantages of the ILLMO environment are the following:

- a greater variety of probability distributions to choose from (next to the default Gaussian distribution),
- the method of multi-model comparison that allows to assign likelihoods to alternative models,
- the method of log-likelihood profiles (LLPs) and the construction of (asymptotically correct) approximations to confidence intervals for most parameters in parametric models (using Wilks’ theorem),
- the extension towards empirical-likelihood profiles (ELPs) so that confidence intervals can also be created in case of non-parametric models,
- the possibility to create ROCs for both parametric models (using log-likelihood) and non-parametric models (using empirical likelihood),
- a methodologically sound way of creating parametric (Thurstone) models for ordinal data such as generated by Likert scales.

The same methods introduced here for comparing two experimental conditions can be extended to a range of other statistical tasks such as simple and multiple regression, nonlinear regression, multi-dimensional scaling, etc., as evidenced by the material offered on the project website http://illmoproject.wordpress.com. Obviously, the range of methods offered in ILLMO is more limited than the range of methods offered through a platform such as R, which can boast a huge community of developers and users. The aim of ILLMO is instead to offer access to methods that can be used to analyse frequently used experimental designs, and to do this in a user-friendly and interactive way, so that a wider range of users, especially those not keen on programming, can also profit from such recent statistical methods.

## Footnotes

1 The estimate for *C**I*(*R*) is obtained by assuming that the variable *z*, obtained by means of the Fisher mapping $z=0.5 \log ((1+R)/(1-R))$ is (approximately) normally distributed with standard error $se=1/\sqrt {N-3}$, where *N* denotes the number of observations. The 95% confidence interval *C**I*(*z*) can hence be easily determined and mapped to *C**I*(*R*) by applying the inverse Fisher mapping $R=\tanh (z)$.

2 The power can be shown to be equal to *P*_*n*_(*t* − *t*_*α*_) (in case *t* > 0), where *t* is the observed value in the *t* test and *t*_*α*_ is the threshold value adopted in the *t* test. By assuming that *t* will grow proportionally to $\sqrt {N}$, we can estimate the value of *N* required for a desired power level of e.g. 0.8.

3 Multi-model comparison and Bayes estimation share this ability to assign likelihoods to alternative models for the same data. Despite the fact that the methods used to establish these likelihoods using both approaches are quite distinct, the resulting likelihood ratios are often very similar.
